# Trends in Recently Emerged* Leishmania donovani* Induced Cutaneous Leishmaniasis, Sri Lanka, for the First 13 Years

**DOI:** 10.1155/2019/4093603

**Published:** 2019-04-14

**Authors:** Yamuna Siriwardana, Guofa Zhou, Bhagya Deepachandi, Janaka Akarawita, Chandanie Wickremarathne, Wipula Warnasuriya, Chandanie Udagedara, Ranthilaka R. Ranawaka, Indira Kahawita, Dananja Ariyawansa, Ganga Sirimanna, P. H. Chandrawansa, Nadira D. Karunaweera

**Affiliations:** ^1^Department of Parasitology, Faculty of Medicine, University of Colombo, Colombo 00800, Sri Lanka; ^2^University of California, Irvine, CA 92697, USA; ^3^National Hospital, Colombo 00800, Sri Lanka; ^4^Teaching Hospital, Kurunegala 60000, Sri Lanka; ^5^Teaching Hospital, Kandy 20000, Sri Lanka; ^6^Teaching Hospital, Kalutara 12000, Sri Lanka; ^7^Base Hospital, Homagama 10200, Sri Lanka; ^8^Sri Jayewardenepura General Hospital, Nugegoda 10250, Sri Lanka; ^9^226/1, Robert Gunawardena Mawatha, Battaramulla 10120, Sri Lanka; ^10^332/4, Anagarika Dharmapala Mawatha, Nupe, Matara 81000, Sri Lanka

## Abstract

Sri Lanka reports a large epidemic of cutaneous leishmaniasis (CL) caused by an atypical* L. donovani *while regional leishmaniasis elimination drive aims at achieving its targets in 2020. Visceralization, mucotrophism, and CL associated poor treatment response were recently reported. Long-term clinico-epidemiological trends (2001-2013) in this focus were examined for the first time. Both constant and changing features were observed. Sociodemographic patient characteristics that differ significantly from those of country profile, microchanges within CL profile, spatial expansion, constant biannual seasonal variation, and nondependency of clinical profile on age or gender were evident. Classical CL remains the main clinical entity without clinical evidence for subsequent visceralization indicating presence of parasite strain variation. These observations make a scientific platform for disease control preferably timed based on seasonal variation and highlights the importance of periodic and continued surveillance of clinic-epidemiological and other characteristics.

## 1. Introduction

Leishmaniasis is considered as a neglected tropical disease [1, 2]. Out of many virulent species in the genus,* L. donovani* is probably the worst and the causative agent of visceral leishmaniasis (VL). Estimated annual VL incidence in developing countries amounts to 200* *000–400* *000 cases [3]. Both cutaneous leishmaniasis (CL) and VL have shown increasing global trends in the recent past [3, 4]. Understanding the epidemiology of leishmaniasis infections and new clinical patterns are useful for disease management and epidemic control.

Leishmaniasis remained rare and mainly is imported in nature until 2001 in Sri Lanka, when autochthonous CL was detected in a patient referred to author's institution during an attempt to identify a parasitic aetiology. A series of professional and public awareness programs (2001-2003) were subsequently carried out by the investigators, which probably resulted in the detection of many more cases from the same region in Northern Sri Lanka. Investigations pointed towards already established local transmission cycle [5].

Initially reported cases were consistent with a clinical picture of CL with no clinical or immunological evidence for visceralization (negative formol gel screening or rK39 rapid assay and absence of systemic features) [5]. Patients presented with papules, nodules ulcerating nodules, or completed ulcers [5]. Atypical manifestations of CL were extremely rare [6]. Causative species was identified as an atypical genetic variant of* L. donovani* [7, 8].

During the subsequent years, spatial widening, case clustering, and peri-domestication were evident [9–12]. VL and mucosal leishmaniasis (MCL) were also reported subsequently [13–15]. Studies suggested a genetic basis for the different local phenotypes [16]. Meanwhile 34% of active CL infections showed a humoral response though follow-up studies have failed to detect any visceralization of initial CL [17, 18]. The felt local need for disease containment prior to the establishment of more virulent forms was highlighted [19, 20].

Regional drive on leishmaniasis control aims at eliminating VL in the Indian subcontinent (ISC) by 2020 [21]. However, predictions suggest that* L. donovani *transmission will continue in this region even after 2020 necessitating the careful surveillance and control [22]. Based on the general belief of the absence of animal reservoir hosts for* L. donovani*, reduction of human reservoirs is considered useful in this regard [23]. Detailed understanding of local disease trends and efforts on enhancing case detection are therefore urgently necessary for disease control. Local leishmaniasis clinical profile has been studied using smaller patient populations spanning a few years during the current outbreak [5, 10–12, 24, 25]. Clinical and spatial trends of leishmaniasis have not been investigated to any level of depth in this focus. Meanwhile increasing number of case referrals to central laboratory in author's institution was observed until decentralization of diagnostic facilities by the same in 2014 and increasing case numbers continued to be reported at hospital settings even after that. Therefore, a relook at the current profile and study of trends of leishmaniasis situation are warranted in this country.

## 2. Materials and Methods

Details from 1953 patients referred to the Department of Parasitology, Faculty of Medicine in University of Colombo (UCFM), over a period of 13 years (2001 -2013) with suspected leishmaniasis, were included in the analysis following informed written consent. Subjects were clinically assessed by the principal author or medically qualified assistants on suspected cases. Sociodemographic and systemic clinical data was gathered using interviewer administered case record forms. Lesion data were collected for each lesion from all suspected CL cases. In case of multiple lesions, randomly selected first lesion was evaluated.

Bone marrow (BM) and lesion material (lesion aspirates, LA; slit skin scrapings, SSSs; punch biopsies, PB) were obtained from suspected cases of VL and CL, respectively. Light microscopy (LM) was carried out on all BM, LA, and SSS samples.* In vitro* cultivation (IVC) of* Leishmania* parasites was carried out using BMs and LAs of LM negative patients [26]. PCR was performed on LM and IVC negative patient's BM, LA, and PBs [27]. Two millilitres of intravenous blood was drawn from all patients and sera were separated. A proportion of laboratory confirmed cases of CL were screened by formol gel test (FGT) (n=700) and rK 39 (n=200) assay, respectively. If a patient was reported positive at least by one parasitological investigation (LM, IVC, and PCR) they were included in the laboratory confirmed group. Those patients who turned negative with all three tests were considered as true negative cases. Inconclusive cases were excluded from analysis. Laboratory confirmed CL cases were considered for sociodemographic and clinical data analysis. Four subgroups were selected from four different time points during the reporting period, that is, A: 2001-2004, B: 2005-2007, C: 2008-2010, and D: 2011-2013. Case referral rates as a projection of case incidence rate (CRR, number of confirmed cases/ 100 000 population) were calculated for each time period in each administrative district within the country.

### 2.1. Data Analysis

Age and sex distribution and clinical presentations were compared between different study periods using *χ*-^2^ test. Maps of spatial distribution patterns were generated using ArcGIS 10.0.

### 2.2. Working Definitions

*Typical Onset.* This is single, painless skin papule of less than 1cm in size.

*Size.* Maximum diameter of the observable lesion is measured to the closest centimetre excluding induration. 

*Main Lesion Stages.* Common lesion types are normally seen during the progression of a skin lesion in CL: a papule of <1cm, a nodule of > 1cm, an ulcerating nodule, or a completed ulcer.

### 2.3. Ethical Aspects

Ethical clearance was obtained from the Ethics Review Committee of Faculty of Medicine, University of Colombo, Sri Lanka.

## 3. Results

### 3.1. General Descriptions

A total of 1,958 patients (75 for VL, 1,883 for CL) investigated in the UCFM during the study period (2001-2013) were studied. VL investigations were carried out on increasing number of individuals since 2005. Out of this 4% (3/75) suspected cases of clinical VL (discussed in Siriwardana* et al. *2017a) [14] and 86.4% (1627/1883) suspected cases of CL were confirmed as positive by laboratory examinations. Confirmed cases of CL are further discussed in sections below.

### 3.2. CL clinical Presentations and FGT/rK39 Examinations

Presence of nonspecific systemic features on examination (fever, loss of appetite, reported loss of weight, splenomegaly, hepatomegaly, anemia, jaundice, and skin colour changes as noticed by patient) was minimal (the highest being 0.3% for loss of appetite and loss of weight) or absent (skin discolouration, 0.0%) among patients with CL. Combinations of any of these features were also minimal (loss of weight and loss of appetite in 0.1%) in the CL patient group. FGT and rK39 screening assay were negative in all examined cases of clinically suspected CL.

### 3.3. CL Spatial Distribution and Trends

**Figure 1:**
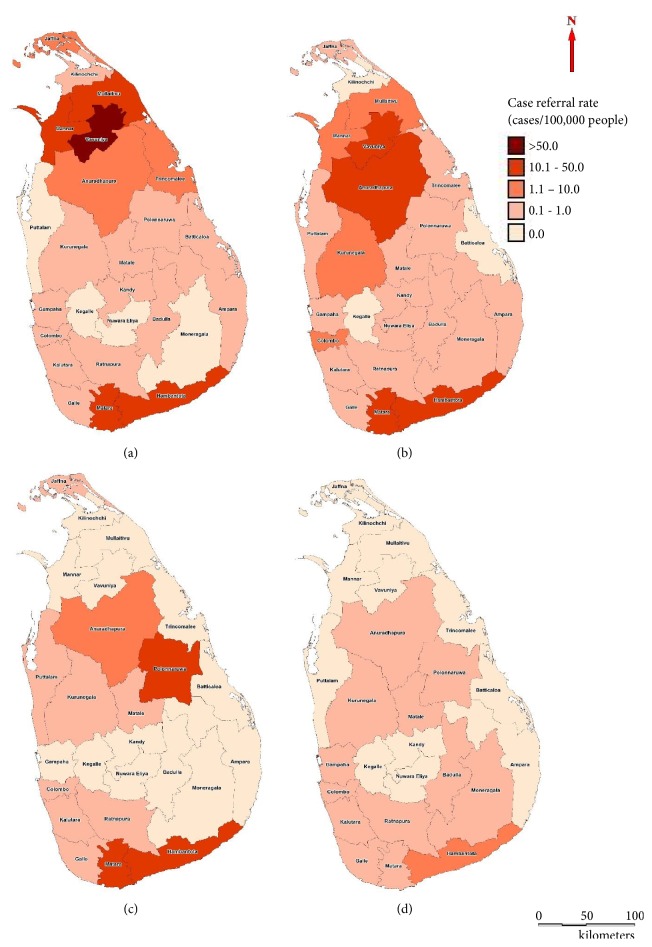
Spatial distribution of passively detected patients with CL in Sri Lanka during the study period (2001-2013). (a) 2001-2004, (b) 2005-2007, (c) 2008-2010, and (d) 2011-2013.

During the early stage (2001-2004), majority of referrals were from 5 administrative districts in Northern and Southern Provinces of Sri Lanka (Figure 1(a)). Most districts (17/25) reported CRR < 1.0 during this period (Figure 1(a)). During the second period (2005-07), CRR remained almost the same; however, incidence in the highest CRR dropped during 2001-04 (Figure 1(b)). During the subsequent periods, CRR showed varying trends. However, increasing number of cases was reported from other regions (Figures 1(b)–1(d)). Cases reported in the previously leishmaniasis prevalent areas in North and South remained almost unchanged over the time (Figure 1). There was a reduction in the total number of cases referred to the institutional laboratory over the years.

### 3.4. Seasonality of Infections

**Figure 2:**
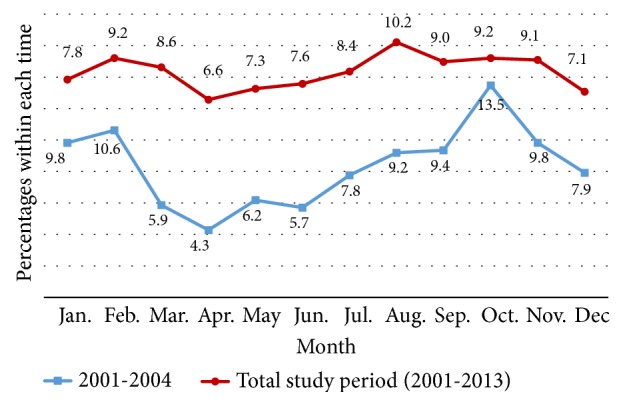
Seasonal variation of CL case presentation in the study population over the epidemic. A low transmission season was observed from March to June and a major high referral season was observed from July to November.

Although reported annual case numbers varied over time, they showed a general seasonal trend; that is, there were a low transmission season from March to June and a major high referral season from July to November (Figure 2). This seasonal variation pattern was more pronounced during the initial stages (2001 -2004), as shown in Figure 2.

### 3.5. Sociodemographic Characteristics

#### 3.5.1. Age and Sex

CL affected all ages, from 1 to 81yrs, regardless of sex. However, analysis of the total laboratory confirmed that CL patient group revealed a significant male preponderance (67.7%, 1102/1627) and involvement of majority of cases between 21 and 40 years (46.6%, n=759) (Table 1). Less than one quarter of cases were young individuals ≤ 20 years (23.3%, n=379). Nearly 1/3 was elderly individuals of >40 years (30.1%, n=489). An approximate 1/3^rd^ of patients were females (32.3%, n=525). Age and sex compositions of patients were significantly different from the Sri Lankan census data (Table 1).

#### 3.5.2. Changes in Demographics

**Figure 3:**
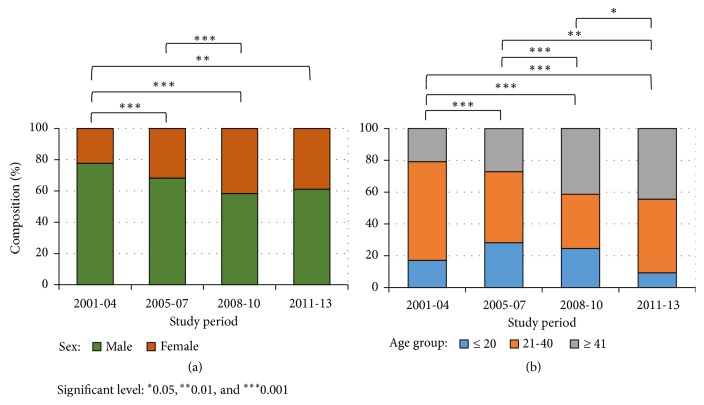
Composition of clinical cases within the study period. (a) Composition of sex, (b) composition of age groups; study period: A-2001-2004; B-2005-2007; C-2008-2010; D-2011-2013.

During the study period, census data showed that the largest difference in sex distribution was male: female 48.2: 51.8% from 2011-13 and census mean sex ratio was male: female 49:51% from 2000 to 2015. However, sex distribution in reported CL cases has never been even close to that. Reported CL cases were always male dominant with a proportion ranging between 58.5 and 77.7%. It varied significantly over time (average 67.7%, *χ*^2^ = 41.8, df = 3, and P < 0.0001) (Figure 3(a)). Similarly, during the study period, census data showed an age distribution of 34.1: 31.2: 34.7 for age groups ≤20: 20~40: >40 yrs. However, age distribution in reported CL cases was 23.3: 46.6: 30.1 for the same age groups, and proportion of cases reported for age >40 yrs increased from 20.8% in 2001-04 to 44.4% in 2011-13 (*χ*^2^ = 98.0, df = 6, and P < 0.0001) (Figure 3(b)). Further analyses revealed that sex and age distributions changed all the time (Figure 3).

#### 3.5.3. Clinical Manifestations

**Figure 4:**
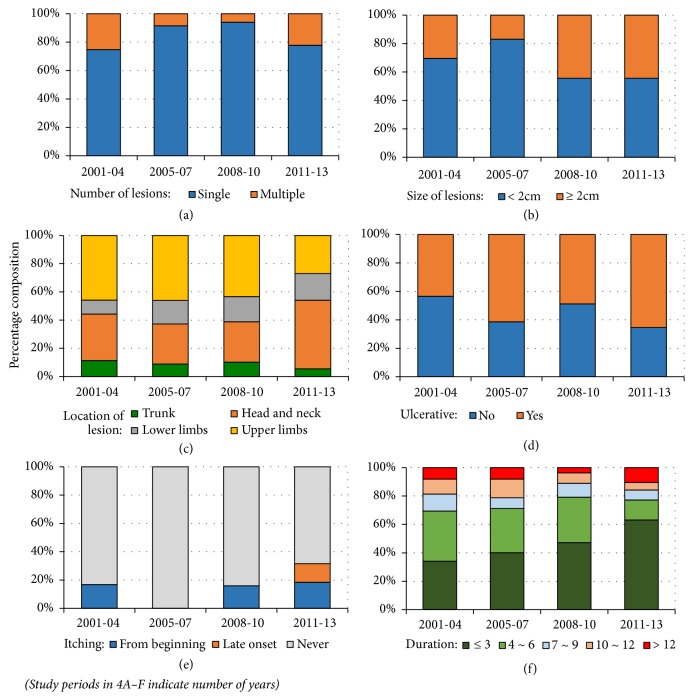
Trends in the clinical profile of study population over time (2001-2013). The individual figures show the variation of (a) number of lesions, (b) size of lesions, (c) location of lesion, (d) type of lesion, (e) itchiness associated with lesions, and (f) duration of lesions.

Majority of lesions reported a typical onset (96.8% or 606/626). Occurrence of a single primary lesion at the beginning was the commonest mode of lesion onset during all stages of the outbreak. Majority of lesions remained single (overall 86.4%, 846/979). However, number of lesions varied over time (range of 74.8~94.0%, *χ*^2^ = 64.9, df = 3, and P < 0.0001) (Figure 4(a)). The majority (65.0% or 672/1034) of the lesions were < 2 cm at the time of laboratory confirmation (Table 2); however, a majority of them were ≥ 2 cm from 2008 and onwards (*χ*^2^ = 51.4, df = 3, and P < 0.0001) (Figure 4(b)). Vast majority of lesions were on exposed body areas (range of 87.6~91.9%) (*χ*^2^ = 1.2, df = 9, and P = 0.749) (Figure 4(c)). Upper limb (44.2%, 463/1047) was the most affected site while trunk was the least affected (10.1%, 106/1047). Both ulcerative and nonulcerative stages of a CL lesion were observed in nearly equal proportions (ulcerative: nonulcerative = 50.5: 49.5%, n = 1028); however, the proportions varied over time (*χ*^2^ = 22.5, df = 3, and P < 0.0001) (Figure 4(d)). Patients experienced lesion associated itchiness in approximate 16.9% (61/332) of lesions at least during a single time period during the course of illness (*χ*^2^ = 107.9, df = 6, and P < 0.0001) (Figure 4(e)). Majority of the lesions presented within the first 6 months of onset/notice all the time (69.4~79.2%), less than 10% presented after 12 months, and the pattern varied during the study period (*χ*^2^ = 39.2, df = 12, and P < 0.0001) (Figure 4(f)). Mean duration of a skin lesion (MLD) was 6.64 months (Table 3). MLD of ulcerated lesions decreased over the time (Table 3).

#### 3.5.4. Age and Sex Cross-Examination

**Figure 5:**
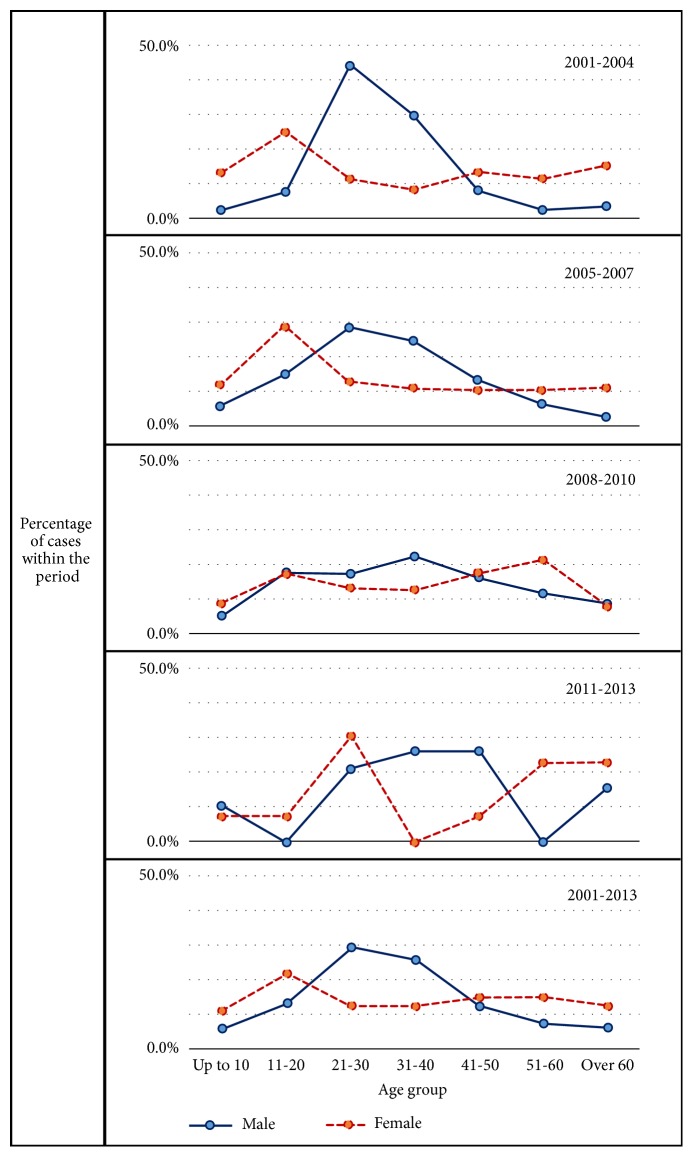
Trends in the sex composition in different age groups over the time. The trend remained nearly constant at different stages during the study period.

More young adult patients (21-40 years) were found among males, while there was a wider age distribution among females with involvement of higher proportions of younger and elderly individuals (Figure 5). This trend remained nearly constant at different time periods during the reporting period (Figure 5). Females were more likely to present with head and neck lesions than males while males had more trunk lesions than those of female group. This difference was seen at all stages of the outbreak. Nature of onset, number, type, or size of lesions did not demonstrate a gender based variation (data not shown). Higher proportion of elderly individuals (>40 years) presented early (51.49% within 3 months) but 53.3% lesions were ulcerated. This trend remained unchanged throughout the reporting time. However, majority of lesions that occurred in all age groups started as primary lesions, remained single and small (<2cm), and were reported early (within 6 months of duration). There was no significant variation in the basic clinical profile between different age categories (data not shown).

## 4. Discussion

Leishmaniasis was made notifiable in the country only in 2009. National data for spatial distribution of reported cases is available since then. However, first leishmaniasis patient diagnostic laboratory was set up at University of Colombo's Faculty of Medicine (UCFM) in 2001 and functioned as the main referral centre for patient diagnosis in the country. Microscopic training provided by this unit for health sector and other institutional technical officers may have led to the decentralization of the diagnostic facilities during the fourth stage (approximately after 2013). This likely has resulted in a drastic reduction of the referred case numbers to UCFM. Until such time, the case referral rates observed at UCFM laboratory could be considered as a near accurate projection of the true case incidence of the country that differ slightly based on the proportion of cases that were not self-referred, misdiagnosed, or treated on clinical grounds due to difficult patient or sample transport to Colombo Laboratory.

**Figure 6:**
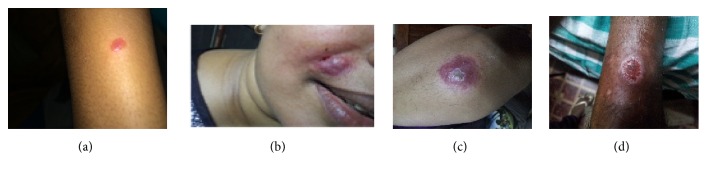
Some common lesion stages in cutaneous leishmaniasis. (a) Popular lesion, (b) nodular lesion, (c) ulcerating nodule, and (d) ulcer.

Clinical leishmaniasis cases in Sri Lanka are still on the rise, with the reported new clinical cases of 1,508 in 2017 by the Ministry of Health [28]. However, the number is relatively small compared to that of dengue fever and other tropical diseases; considering the fact that Sri Lanka is a small country, the increasing trend in* L. donovani *caused CL in Sri Lanka is definitively worrisome with the mission of leishmaniasis elimination in Indian subcontinent. Understanding the epidemiology of leishmaniasis and past control practices is useful for future targeted disease control planning. Findings from this study may well serve that purpose. Main clinical picture of leishmaniasis in Sri Lanka over the study period continued to remain as CL (Figure 6). Other clinical forms of leishmaniasis still remain a minority, though it may be an underestimation of the true picture [14]. Minimal or absent systemic features in a clear majority of CL cases, negative VL screening assay results in CL, absence of an initial history of CL in reported VL cases [14], and already reported strain difference between CL and VL causing local* Leishmania* strains [18] indicate that most local skin infections progress without visceralization. However, early evidence of serological response may be indicative of a potential for visceralization on the other hand, though it can also be due to a transient CL associated seroconversion [17].

Spatial dimensions of the CL outbreak have expanded during the study period. In spite of this, few districts in Northern and Southern Sri Lanka remained the highest case prevalent areas indicating the possibility of existence of independent disease transmission foci in these areas. Case clustering in the South was identified previously [12]. Spatial expansion of existing transmission foci and increased free movements of people between disease foci and other areas resulting in new foci, fast progressing infrastructure developments, and vector abundance confounded by favourable climatic conditions may have contributed to the disease spread in the island. The improved awareness among clinicians, public health personnel, and the general public may also have contributed to improved case detection.

Changes in spatial distribution showed the locations of the transmission foci and how these foci shifted from time to time. In this study, we found that the transmission hotspot in Southern Sri Lanka along the coastline remained unchanged over time. However, transmission hotspots in the Northern part expanded from 2001 to 2007 and then shifted southwards from 2008 to 2010. However, this study only included clinical data collected from University of Colombo. Importance of case notification [29] and utilization of such data for local surveillance [30] has been indicated. It will be important to include the updated national data so that the movement and current locations of hotspots can be updated. Nonetheless, spatial trends of the epidemic will be important in designing future epidemiological surveillance plans and for resource allocation processes in disease control.

Seasonal pattern of transmission is useful in timing disease surveillance and control activities. Seasonal variation of case distribution with two annual peaks that coincides with the monsoon rain patterns in Sri Lanka was evident since the onset of the epidemic seems to remain unchanged over the time. Presence of an established pattern maintained over a decade period indicates the long-term existence of the local infection, which has been backed up through phylogenetic analyses [8, 18, 31]. This is likely the reflection of seasonal activity of vectors. May and September monsoon brings rain to the Southwest of Sri Lanka, while the dry season in this region is from December to March. In the Northern part of the country, the North-Eastern monsoon from October and January brings both wind and rain in that region, and drier weather is between May and September. From 2001 to 2004, the peak transmission season was from August to March, during which most of the cases were from Northern part; that is, they peaked during monsoon season.

CL affected a wider age range (1- 81 years, data not shown) and both genders. However disease associated male and young adult age preponderance observed since the onset of the epidemic was clearly different from the age and sex composition of the country's normal population. Outdoor associated behaviours and occupational exposure are the most likely reasons for the male and young adult age preponderance. Though these patterns remain unchanged over the epidemic, a changing age sex pattern with increased involvement of older age groups and females during the later stages was observed. This may be indicative of an association with behavioural patterns and peri-domestication of transmission cycle that increases the risk of disease being transmitted to them. Peri-domestication was also indicated in previous studies that identified household risk factors [10]. The expansion of transmission from mainly male young adults to female and older age groups may pose a significant threat to disease control activities.

Majority of cases presented early had typical CL lesions (Figure 5). Majority of lesions in the total study population remained as single and small that were seen on exposed body sites. Proportion of chronic (>3months old) lesions increased significantly over time together with increased proportions of large (>2cm) lesions observed later on. However a considerable proportion of lesions were either presented or detected over a year from the onset/notice of the skin lesion indicating the need for improved case suspicion or early diagnosis. Common involvement of exposed areas is consistent with the common clothing patterns that expose the affected sites to the sand fly vector. CL lesions in a clear majority may occur on the site of vector bite as single lesions indicating minimal cutaneous spread. Itching was not a widely recognized known feature associated with leishmanial skin lesions. However, since a good minority of skin lesions were associated with itchiness, suspected CL lesions should not be exempted as non-CL and therefore from proceeding to laboratory confirmation merely due to presence of itchiness.

Earlier described broad CL profile still remains applicable/undisturbed, though there were minor fluctuations between different time periods of the epidemic. Underlying reasons for this observation may be the noninfection associated factors like changing case referral patterns. Study of data that were collected through active case detection is required for better understanding.

Current or previously observed patterns of clinical features were not shown to be gender dependent except for the differences seen in affected body sites probably due to the clothing patterns. Clinical profile was not age dependent as well, except for the lesion progression which seems to be faster in elderly individuals. Increase in number of single and early lesions may be indicative of better awareness and case detection rates though this may also indicate rapid progression of skin lesions which promote early treatment seeking behaviours.

CL still remains as the main clinical entity with few cases from other clinical entities. These observations further support the existence of multiple genetic variants that cause skin and visceral/mucosal infections, a phenomenon that has already been demonstrated [18]. Though major sociodemographic changes are not observed, minor and continued changes are observed. Likely reasons are peri-domestication, increased host immunity, spatial expansion, improved awareness, or a combination of many factors. Main disease foci are still reported in resource limited areas in the local setting [15, 25]. The observed reduction in self-referral time is encouraging, though there were some chronic lesions. Accurate clinical suspicion especially in a laboratory resource limited setting is required to enhance case detection in new disease foci. Already reported other clinical forms caused by* L. donovani *in local population may further complicate the disease control activities unless they are launched in an evidence based and a timely manner. Dependency on passive case detection is a limitation in this study.

## 5. Conclusions

It is important to carry out periodic surveillance to understand the changing trends within the existing picture and they will provide more accurate projections on the true case burden and epidemiology in the island.

## Figures and Tables

**Table 1:** Summary of clinical cases reported (2001-2013).

Parameter		Case incidence	Composition (%)	Composition from census (%)
Total cases		1,627		
Sex	Male	1,102	67.7	49.0
	Female	525	32.3	51.0
Age (years)	≤ 20	379	23.3	34.1
	20~40	759	46.6	31.2
	> 40	489	30.1	34.7

**Table 2:** Trends in the clinical profile over the study period.

Clinical parameter	Total*∗*		Chi	df	P value
	No.	(%)			
Single	846	86.4	64.9	3	<0.0001
Multiple	133	13.6			
Total	979	100.0			

Exposed area	893	88.7	1.2	3	0.74887
Covered areas	114	11.3			
Total	1007	100.0			

Less than 2cm	672	65.0	51.4	3	<0.0001
larger	362	35.0			
Total	1034	100.0			

Nonulcerative	509	49.5	22.5	3	<0.0001
Ulcerative	519	50.5			
Total	1028	100.0			

Up to 6months	764	74.1	39.2	12	<0.0001
7-12 months	200	19.4			
Over 12	67	6.5			
Total	1031	100.0			

Lesion associated itchiness at anytime	61	18.4	107.9	6	<0.0001
Never	271	81.6			
Total	332	100.0			

*∗*Missing cases or variables were excluded.

**Table 3:** Trends in mean durations of lesion according to selected clinical features.

Feature	Group A 2001-2004	Group B 2005-2007	Group C 2008-2010	Group D 2011-2013	Total group
MLD*∗* (SD) skin lesions	7.7 (8.8)	6.9 (5.9)	5.2 (5.4)	7.0 (9.8)	6.64 (12.3)
MLD (SD) of single lesions	7.5 (7.6)	7.06 (6.1)	5.2 (5.4)	6.2 (6.7)	6.4 (12.5)
MLD (SD) of <2cm lesions	6.7 (8.2)	6.9 (5.7)	5.3 (5.8)	6.2 (6.7)	6.2 (6.5)
MLD (SD) of ulcerated lesions	8.7 (8.9)	7.3 (5.7)	4.9 (5.4)	6.2 (8.0)	6.0 (6.1)

*∗*MLD (SD): mean lesion duration in months (standard deviation).

## Data Availability

Data has not been made available as it was not part of the ethics application and due to patient confidentiality.

## Supplementary Materials

Supplementary Materials Summary of observations is obtained for trend analysis in recently emerged* L. donovani *induced cutaneous leishmaniasis in Sri Lanka for the first 13 years.
