# Supplementary material for: Trends in Recently Emerged Leishmania donovani Induced Cutaneous Leishmaniasis, Sri Lanka, for the First 13 Years
Source: Biomed Res Int. 2019 Apr 14;2019:4093603. doi: 10.1155/2019/4093603 (PMC6487155; doi:10.1155/2019/4093603)
Supplement: Supplementary Materials — Summary of observations is obtained for trend analysis in recently emerged L. donovani induced cutaneous leishmaniasis in Sri Lanka for the first 13 years. [file 4093603.f1.pdf]

Supplementary Materials

Summary of observations obtained for trend analysis in recently emerged *L. donovani* induced cutaneous leishmaniasis in Sri Lanka for first 13 years

**Factors under consideration**	**Initial observation**	**Trends**
Systemic features*	Negligible	Same pattern continued
Lesion associated itchiness	Seen in a minority	Same pattern continued
Onset of skin lesions#	Typical onset in a majority	Same pattern continued
Gender composition	Male preponderance	Same pattern continued
Age and gender comparison	Wider age range in females as compared to male	Same pattern continued
Clinical profile	Mostly single, size >2cm, on exposed body areas	Same pattern continued
Spatial distribution	Majority from leishmaniasis prevalent areas	Same pattern continued
Gender basis for clinical features *	Not observed	Continued
Age based variation in lesion features*	Not observed	Continued
**Important trends observed over the time;**		
Increasing infection among elderly population (>60 years)		
Increasing infection among females		
Widening of affected age range*		

*Data not shown
